# Embedding an economist in regional and rural health services to add value and reduce waste by improving local-level decision-making: protocol for the ‘embedded Economist’ program and evaluation

**DOI:** 10.1186/s12913-021-06181-1

**Published:** 2021-03-06

**Authors:** Andrew Searles, Donella Piper, Christine Jorm, Penny Reeves, Maree Gleeson, Jonathan Karnon, Nicholas Goodwin, Kenny Lawson, Rick Iedema, Jane Gray

**Affiliations:** 1 Hunter Medical Research Institute, University of Newcastle, Callaghan, Australia; 2 New South Wales Regional Health Partners, Newcastle, Australia; 3 School of Medicine and Public Health, University of Newcastle, Callaghan, Australia; 4 School of Rural Medicine, University of New England, Armidale, Australia; 5 Hunter Medical Research Institute, New Lambton Heights, Australia; 6 Faculty of Health and Medicine, School of Biomedical Sciences & Pharmacy, University of Newcastle, Callaghan, Australia; 7 College of Medicine and Public Health, Flinders Health and Medical Research Institute, Flinders University, Adelaide, Australia; 8 Faculty of Health and Medicine, Central Coast Research Institute for Integrated Care, University of Newcastle & Central Coast Local Health District, Gosford, Australia; 9 Centre for Team-Based Practice & Learning in Health Care, King’s College London, London, UK; 10 Partnerships, Innovation and Research, Hunter New England Local Health District, New Lambton, Australia

**Keywords:** Health economics, Economic evaluation, Program evaluation, Measuring impact, Embedded researcher, Health services research, Value-based healthcare

## Abstract

**Background:**

Systematic approaches to the inclusion of economic evaluation in national healthcare decision-making are usual. It is less common for economic evaluation to be routinely undertaken at the ‘local-level’ (e.g. in a health service or hospital) despite the largest proportion of health care expenditure being determined at this service level and recognition by local health service decision makers of the need for capacity building in economic evaluation skills. This paper describes a novel program – the embedded Economist (eE) Program. The eE Program aims to increase local health service staff awareness of, and develop their capacity to access and apply, economic evaluation principles in decision making. The eE program evaluation is also described. The aim of the evaluation is to capture the contextual, procedural and relational aspects that assist and detract from the eE program aims; as well as the outcomes and impact from the specific eE projects.

**Methods:**

The eE Program consists of a embedding a health economist in six health services and the provision of supported education in applied economic evaluation, provided via a community of practice and a university course. The embedded approach is grounded in co-production, embedded researchers and ‘slow science’. The sites, participants, and program design are described. The program evaluation includes qualitative data collection via surveys, semi-structured interviews, observations and field diaries. In order to share interim findings, data are collected and analysed prior, during and after implementation of the eE program, at each of the six health service sites. The surveys will be analysed by calculating frequencies and descriptive statistics. A thematic analysis will be conducted on interview, observation and filed diary data. The Framework to Assess the Impact from Translational health research (FAIT) is utilised to assess the overall impact of the eE Program.

**Discussion:**

This program and evaluation will contribute to knowledge about how best to build capacity and skills in economic evaluation amongst decision-makers working in local-level health services. It will examine the extent to which participants are able to improve their ability to utilise evidence to inform decisions, avoid waste and improve the value of care delivery.

**Supplementary Information:**

The online version contains supplementary material available at 10.1186/s12913-021-06181-1.

## Background

### The need for economic evaluation in healthcare

Determining whether healthcare spending choices represent value for governments, patients and taxpayers depends on whose perspective is taken, but in general ‘value’ can be assessed by examining the impacts of spending choices on health service efficiency and equity. Decisions are made daily in healthcare about the type of care that will be provided, not only to individual patients but also to improve the health and wellbeing of all people living in local communities. These decisions can be about new medicines, new technologies, improved models of care or approaches designed to promote health and prevent or manage avoidable illnesses within the population.

Choices must be made as to what will and won’t be provided by the health system. Ideally, these choices should be made on the best available evidence, using a value-based framework that considers the likely impact on people’s care experiences and outcomes to the investment that is required to achieve them. Such decisions should consider the resource use associated with each course of action, not just for providing a new technology or model of care, but also the impact the decision has in future time periods. For example, new drugs to reduce cholesterol will have ‘a purchase price’ but they also reduce the risk of some cardiovascular events in future years, and hence they contribute to reducing resource use associated with future hospitalisations and interventional procedures. Case management of patients with complex chronic illnesses is another example: this may support reduced hospitalisations, but does require significant long term investment in the development of the primary and community care system. While some impact could be seen quickly, maximum impact is likely to be in the future and many confounders will limit direct attribution of health gains to the new model of care.

In the absence of necessary evidence, decision makers may have little idea of the outcome from investing in a technology, model of care or policy. The lack of information about clinical and cost-effectiveness of existing technologies, models of care and health policies has been linked to economic waste in health care in Australia [1]. That is, without economic evaluation we do not know whether a health technology provides patients with benefit and we may not have visibility on the resource use associated with a technology. Hence, part of the $185 billion we spend on healthcare in Australia annually (accounting for about 10% of the country’s economic activity [2]) is funding health care that has unknown benefit – and some of this unevaluated healthcare will not only have no benefit; it may well cause harm [3, 4].

For some nationally remunerated health technologies – such as prescription medicines – Australia has clearly articulated and legislated guidelines for the evaluation that *must* be undertaken [5]. New medicines are continuously being listed on the Australian Pharmaceutical Benefits Scheme (PBS) and for each new listing, the Government has good evidence of both the cost and the benefit of each medicine they are subsidising. However, consideration of healthcare provided at what we refer to as the ‘local level’, such as in hospitals, primary care, and local health districts or networks, shows that economic evaluations of technologies, models of care and policies are not systematically undertaken. When they are undertaken, the methods used vary in appropriateness and quality and results may not be easily understood or translatable [3].

Healthcare spending at the local level accounts for the largest share of total health spending in Australia [3]. It is not only the *largest* share of health spending; it is also the *fastest growing* within the health portfolio [2]. Compared to the previous year, the fastest increases in healthcare spending in 2017–18 were for hospitals (public and private), accounting for $74 billion, and primary healthcare, accounting for $63 billion [2]. Hospital and primary care spending accounts for 38 and 28% of total health expenditure respectively, and in the 10 years from 2005/06 to 2015–16 spending on hospitals rose, on average, by 7.5% per annum and spending on primary care grew by 6.4% per annum [6].

Just spending more money on healthcare does not guarantee better health outcomes. It is neither a solution to Australia’s emerging healthcare needs nor is it sustainable. Governments, health services, patients and taxpayers want to know the value that is being delivered by every health dollar [3]. With the advent of the COVID-19 pandemic and rising public debt, the need for understanding value from healthcare spending has increased [7].

Given the substantial proportion of healthcare funding managed at the local level, it is surprising that more attention is not placed on economic evaluations of the technologies, models of care and policies that are supported at this level. Indeed, when new and potentially complex service innovations – such as integrated health and social care are piloted as means to address increasing demands on the local health system, it is most often the case that evaluation of their costs and impact is either missing or poorly applied [8, 9]. One reason for this may be the complex and challenging environment in which local health services operate: evidence-based investment and disinvestment decisions may not be easy to apply in a health system with both Commonwealth (primary and aged care) and State (hospital) funding streams and different cost centres operating at the local level. Also, there is a need to involve clinical leaders in its production of local evidence so that they are more inclined to act upon its results. This requires capacity building in evaluation and collaborative decision making between health service managers and clinicians.

### Addressing the local level evaluation challenge

Research undertaken in Australia in 2018 [3] took up the challenge put forward by the Australian Productivity Commission [1] and focused attention on the problem Australia has with the *local level evaluation* of healthcare. In 2018, New South Wales Regional Health Partners (NSWRHP), an Australian translational research centre accredited by the National Health and Medical Research Council (NHMRC) and funded by health and research partners and the Medical Research Future Fund (MRFF), produced a National Report under the auspices of the Australian national alliance of health research translation centres (Australian Health Research Alliance (AHRA)) [3] .Work including a literature review and in depth interviews with health service managers across Australia, was overseen by an expert panel, including representatives from health services who brought operational perspectives to interpretation of results and development of recommendations.

This research revealed that local health services “were starved for evaluation staff and evaluation skill-sets” [3]. In terms of solving the economic evaluation problem, data from health services highlighted that the development of *internal* capacity and capability in evaluation, that includes economic skills, was the preferred direction [3]. Health services indicated they wanted experts who would co-locate with staff in local health services and work with them to co-design evaluations. Health services which had prior experience commissioning evaluations (and economic evaluations in particular) from third parties such as commercial consultancies or academics, expressed dissatisfaction with both the process and the quality of the outputs of these evaluations. Examples were cited where external academic evaluators had prioritised their own research interests ahead of directly tackling the evaluation problems posed by health services [3]. The consultations also showed that there was a strong interest from health services in developing in-house evaluation expertise in general and in-house evaluation capacity specifically in health economics [3].

The recognition of the need for health economics in operational health services is not new. Earlier research by Ross [10] examined senior government managers’ (including health care managers) attitudes to economic evaluations, finding that there was a high level of awareness of the value of economic evaluation. However, managers considered their lack of economic evaluation expertise and knowledge was a major barrier to access, which was compounded by a scarcity of health economists in Australia [10]. Other barriers to using health economics in decision making included poor communication due to, for example, unnecessary use of economic jargon and it was noted that some academic health economists tended to place more emphasis on the rigour of their methods than on communicating the principles involved to the decision makers [10].

Based on the literature review, in depth qualitative investigation and insights from the expert steering committee, the National Report [3] recommended approaches for increasing economic evaluation skills at the local-level. The four major recommendations were to support capacity building through:

1. Establishing a national Expert Panel of people with the skills to develop the national and local level health evaluation and implementation framework (the research developed a model framework) and a National Advisory Committee on Health Evaluation and Implementation.
2. Boosting education and training, and professional development for clinicians and managers, to ensure a health services workforce that is ‘evaluation and implementation capable’.
3. Increasing the workforce of skilled evaluation staff at the local level (in health services and affiliated organisations), particularly in health economics.
4. Facilitating an increase in evaluation and implementation resources at the local level to support a sustainable integration of evaluation and implementation capability into health services’ decision making [3].

In response to these recommendations, NSWRHP and Health Translation SA developed a pilot intervention, (which addresses recommendations 2, 3 and 4), consisting of:

- Capacity and capability building in local health services via co-location of rare evaluation expertise (health economics) *within* six local level health services and
- Targeted and facilitated ‘purpose designed’ education, training and support in evaluation methodologies, including health economics [3].

Together, these two elements are herein referred to as the *“embedded Economist”* (eE) program.

### Conceptual underpinnings of the eE program

The approach of embedding health economics expertise into an operational health services is supported by prior work: Specifically the premise for embedding is to enable the health economists to engage with health services’ staff to better understand their questions and concerns, rather than focusing on a more academic and transactional approach to undertaking research evaluations [10–18]. Major advantages of embedding health economics expertise into an operational health service include the ability to build trusting relationships, leading to better understanding of local context, the services’ aims and pressures; the ability to tailor strategies accordingly; and to direct feedback to improve implementation of outcomes [18, 19]. Immersion also enables capacity building as the health economist can teach skills that are directly relevant to staffs’ everyday work situations while immersed and health service staff provide the opportunity for health economists to connect with real world issues and challenges [19].

The detailed conceptual underpinnings of the eE program are reported elsewhere (under review). By way of summary, the embedded approach is grounded in co-production [20], embedded researchers [18] and Stenger’s concept of ‘slow science’ [21]. Slow science is a metaphor for embedding the process of research with those who would use or benefit from it so that the findings are attuned to the dynamics and complexities that define health service delivery. The eE program charges the health economist to connect with local problems and health service staff on their terms, and frame findings and solutions in ways that resonate with, and are meaningful to, an operational health service.

The eE educational intervention has been designed around a Community of Practice (ComPrac) model to facilitate the social and organizational learning implicit in the embedded approach [22] . Communities of Practice (ComPracs) are groups who engage explicitly in collective learning in a shared domain. They have a passion or common interest in something they do (this gives them an identity), and they interact regularly in order to learn to do it better - this makes them cohesive [23]. The eE ComPrac will enable learning support that facilitates social interaction and collaborations and networks, in order to share expertise and increase exposure to the application of knowledge [22].

## Methods

### Aims of the eE program

The aims of the eE Program are:

1. To increase health service staff awareness of the value economic evaluation can bring to decision making.
2. To develop health service staff knowledge and capacity to access and apply economic evaluation principles, methods and tools in decision making through formal training and extended exposure to an embedded economist.
3. To facilitate health service practice change and the routine application of economic evaluation principles in decision making.

Further aims of the study are to *evaluate* the contextual, procedural and relational aspects of embedding an economist within health service and capture the outcomes and impact of the program.

The study hypothesis is that embedding a health economist and providing education within a health service will result in:

1. Increased staff awareness of the benefits of economic evaluation
2. A developing capacity to access and apply economic evaluation principles, methods and tools
3. Outputs in the form of tools to assist in conducting economic evaluations and economic evaluations of services, technologies, programs and policies
4. Emerging use of these outputs to inform decision making
5. Benefits greater than costs for each health service research site
6. Benefits greater than costs for the overall program

### Sites, researchers and participants

Five New South Wales Regional Health Partners (NSWRHP) sites agreed to participate in the eE program:

- A pilot site: Hunter New England Central Coast Primary Health Network (HNECCPHN). Piloting the program in this site enabled the intervention and study protocol to be refined.
- Four subsequent NSW sites:
  - Mid North Coast Local Health District (MNCLHD)
  - Central Coast Local Health District (CCLHD)
  - Hunter New England Local Health District (HNELHD)
  - Calvary Mater Newcastle Hospital (CMN)

One inter-state site, Southern Adelaide Local Health Network (SALHN) in conjunction with Health Translation South Australia (HTSA) will also implement the embedded Economist program.

Implementing the embedded Economist program and studying outcomes in several Australian state health systems (public and private) will greatly accelerate translation of positive findings nationally. There is now a developing Australian special interest group of highly-applied health economists meeting under the umbrella of The Australian Health Research Alliance (AHRA), which will in part address Recommendation 1 from the National Report [3].

Given that the principles of co-production, embedded research and ‘slow-science’ underpin the program, boundaries between researchers and health service participants are blurred: A core team of researchers is made up of a *Program Lead/health economist* at each site and three *Social Scientist Evaluators.* This core team is complemented by a New South Wales lead economist and South Australian lead economist; overseeing several embedded health economists, all of whom are both researchers and participants. The lead and embedded economists co-produce projects at each site and participate in the evaluation. A *Site Lead* is appointed by the program sites’ executive management. Site leads also occupy dual roles as researchers and participants: they lead the site specific ethics applications; ensure executive endorsement and engagement in co-producing the scope of the site project; manage and administer the embedding economist and recruitment of relevant staff into the education component of the program; but also participate in the program and evaluation as participants by enrolling in the education component or working with the embedded economist on projects.

### Program overview

Two components of the *eE program* will be implemented in the project sites:

#### Embedded economist component

A health economist is located within each site for approximately 3 months per site. In NSWRHP sites, a team of six economists from Hunter Medical Research Institute (HMRI) are participants. They include three senior economists who will share the role of being the nominated embedded economist in each of the five NSWRHP sites (i.e. two leads will each embed at two sites each and one lead at one site only). This senior role is supported by other economists on the team who may or may not be physically embedded in the health service but will support the evaluations co-designed with the health services (e.g. with specialist skills such as advanced modelling).

Being ‘embedded’ requires physical location at the health service between 2 to 3 days per week. While embedded, the economist sits in an area (or different areas depending upon the size of the health service) that are accessible to health service staff – to invite ‘corridor’ conversations and questions about how health economics can assist the health service.

#### Education component

A supported online learning component consisting of:

- a community of practice (ComPrac) and
- a university course on economic evaluation.

Individual staff members may therefore choose to participate in various ways by doing one or more of the following:

- *Connecting* with the embedded Economist at their site by attending workshops and presentations or having a meeting or receiving advice; and /or
- *Co-producing* with the embedded Economist by planning the intervention and/or completing projects as agreed between the economist and participant and set out in site operational plan and/or
- *Engaging* in the online community of practice; and
- *Enrolling* in a university course on health economics and finance.

### Design

#### The ‘embedded economist’ component

The embedded Economist component of the intervention is structured into three phases: pre-implementation (*planning*), implementation (*intervention*) phase and a post intervention (*finalising*) phase. See Table 1 below.

**Table 1:** Overview of the ‘embedded Economist’ component

PHASE	TIMING	ACTIVITIES, OUPTPUTS & TIME COMMITMENT	PURPOSE	PARTICIPANTS
Pre-implementation *(planning)*	3 months prior to embedding 1 month prior to embedding	Initial scoping meetings to develop draft operational plan (minimum 2 × 1 h meetings) Co-production meetings to draft operational plan (minimum 2 × 1 h meetings)	To introduce the project and understand the context, priorities and expectations of the site To begin scoping out a program of ‘placement work’ for the embedded Economist	Lead Economist (*N* = 1) Embedded Economist (*N* = 1–5) Site Lead (nominated by each site) (*N* = 1) Chief Executive and/or nominees (*N* = 5)
Implementation (program)	2 weeks prior to eE embedding	Introductory Education Seminar on health economics and the intervention (2 h)	To begin capacity building and introduce the intervention as well as the Site Lead, Economists and Evaluator to staff beyond those engaged in the planning phase	Lead Economist (*N* = 1) Embedded Economist (*N* = 1–5) Site Lead (*N* = 1) Evaluator (*N* = 1) Current or emerging executives or managers who makes decisions to improve the value of the programs, services, initiatives or technology for which they are responsible
	During the first week of economist embedding	Launch event (a morning tea and/or a meet and greet/presentation) (1 h)		
	During the first two weeks of economist embedding	Draft operational plan refined and finalised Embedded economist allocated to site (if not already allocated) (minimum 2 × 1-h meetings)	To finalise scope of the site project: The tasks, roles and placement of the embedded Economist will be co-designed with each research site	Lead Economist (*N* = 1) Embedded Economist (*N* = 1–5) Site Lead (*N* = 1) Chief Executive and/or nominees (*N* = 5)
	3 months per site as per program timeline	Economist embeds in site, supervised by Lead Economist, and delivers economic evaluation services as agreed in site operational plan. Economist may access the services of the Assisting Economists to analyse data and conduct evaluations whilst embedded. (Minimum participant commitment 15 min (for a brief one-off consultation with the economist) through to a maximum commitment of 100 s of hours. The maximum commitment will depend upon the scope and number of projects co-designed by the site and embedded Economist, which will be set out in the operational plan).	To build capacity in economic evaluation	Lead Economist (*N* = 1) Site Economist (*N* = 1–5) Site Lead (*N* = 1) Current or emerging executives or managers who makes decisions to improve the value of the programs, services, initiatives or technology for which they are responsible (*N* = up to 150 per site)
Post- implementation *(finalising)*	Month following eE leaving site	Closing ceremony (approx. 1 h)	To celebrate achievements and discuss lessons learnt and sustainability of program.	Lead Economist (*N* = 1) Site Economist (*N* = 1–5) Site Lead (*N* = 1) Evaluator (*N* = 1) Current or emerging executives or managers who makes decisions to improve the value of the programs, services, initiatives or technology for which they are responsible (*N* = max 150 per site)
	Within 3 months of eE leaving site	Dissemination of Individual site reports	To provide results for site to participants	Produced by the researchers. Where appropriate links will be provided on the program website.

#### The education component

The education component consists of supported learning in the form of:

- A 12 month online facilitated community of practice: titled ‘*the embedded Economist ComPrac*’. ComPracs can support a large number of participants across different engagement levels: people can opt in and out of facilitated discussions that suit them and there are no mandatory attendance or submission requirements: The time commitment is dictated by the participants and may range from 1 h to 130 h over 12 months.
- A three-month online university unit of study (or ‘Course’) titled: ‘*Health Economics and Finance*’ provided by the University of Newcastle. It will be available free of charge to a maximum of 60 staff across participating sites who have participated in the ComPrac. The course contains topics covering: an introduction to conceptual frameworks and principles of health and healthcare economics; health insurance and financing; healthcare systems design and organisation; an introduction to health economic evaluation; and equity and socioeconomic disparities. The time commitment is 120 h.

The university course provides the theory and tools to apply economic evaluation in a health setting. The ComPrac builds capacity by providing examples and coaching from expert economists in applying economic evaluation skills and tools, and by facilitating networking with others both within and across participating sites.

The COVID-19 pandemic led to a delay in the commencement of the educational intervention, which started in September 2020. This means that the ComPrac and Course members will consist of a mix of health staff who have already experienced the embedded economist on site and completed their site evaluations, those who have the economist currently on site and those preparing to receive the economist.

Calls for expressions of interest in the ComPrac are disseminated by Site Leads, via email, as an attachment to the agenda of the Senior Executive Team, and other meetings as decided by the site, inviting staff members of those committees to consider enrolling and to invite *“current or emerging executives or managers who make decisions to improve the value of the programs, services, initiatives or technology for which they are responsible”.* This flexible approach to participants was deliberate: We wanted sites to have the ability to target not only their current decision makers but also emerging managers, as well as specific pockets of decision makers where the site felt capacity building in economic evaluation was most needed. On enrolment, staff are required to declare that they have their manager’s support to undertake the ComPrac. In early 2021 the Program Manager will email ComPrac participants, asking if they wish to also enrol in the university course and liaise with the Site leads and university to enrol the staff as free students in this course.

Based on resources and conversations with each Site Lead and Executive we expect 60 participants to enrol in the university course and up to 200 participants in the ComPrac.

### Evaluating the ‘embedded economist’

The evaluation consists of site-specific and overall program evaluation. For each site, the following will be assessed pre, during and post program implementation by the Social Scientist Evaluators:

- *Processes* involved in each stage of the eE program at each site: What was done (by whom and with whom)? How it was done, did it work or not work?
- *Context* in which the eE program took place at each site: What contextual factors impacted on each phase at each site?
- *Relational aspects* of each phase of the project intervention*:* Who engaged in each phase at each site? How? What happens when staff and economists are confronted with a different way of thinking and working? Does this result in practice change or is the embedded economist utilised in a transactional way?
- *Outcomes:* does the value staff place on economic evaluations, and their confidence and application increase as a result of the eE program?

These questions will be addressed via the site-based and overall components of the evaluation. Site-based evaluation consists of:

- Non-participant observations
- Documentary analysis
- Semi-structured interviews
- Economist field diaries
- Baseline survey
- A pre and post education component survey

Complementary data will be collected at each phase of the project to capture deep insights into the context, processes, relational aspects and outcomes of the intervention. Our study design adopts methodological triangulation by comparing and contrasting the different data sources. For example, the field diaries provide data of what the embedded Economists do and think. Interviews allow these and other issues identified by the researchers to be explored in more detail, with both economists and staff participants. Observations then enable the researchers to identify if what people say in interviews and field diaries matches what is done in real-time.

Table 2 below describes the evaluation components including participants, tools utilised at different stages and justification for the information being sought.

**Table 2:** Evaluation overview

TOOL	STAGE OF INTERVENTION	DATA SOURCE & COLLECTION	WHO PARTICIPATES	EXPECTED No. OF PARTICIPANTS	TIME COMMITMENT	INFORMATION NEEDED & JUSTIFICATION
Baseline Survey* (detail below table)	PRE & DURING	Online anonymous survey implemented via the ComPrac by the Social Scientist	Current or emerging executives or managers from all sites who enrol in the CompPrac and make decisions to improve the value of the programs, services, initiatives or technology for which they are responsible	80–120 across all sites	15 min	Baseline measure of individual values and confidence and organisational support and barriers around economic evaluation; as well as expectations about the embedded Economist online learning support. This survey will inform the learning support and the remaining embedded components of the program
Non-participant observations	PRE & DURING	Observation notes collected by Social Scientist	Economists and staff who planned or participated in the embedded Economist component of the intervention	Maximum 50 per site	PRE: 5 h maximum: 3 × 1 h scoping meetings 2 × 1 h co-production meeting DURING: a maximum of two hours per interaction for no more than four (4) meetings (minimum commitment 2 h maximum commitment 8 h)	Naturally occurring in situ group data about the process of the intervention (what was done by whom?), the relational aspects (how did the participants interact with one another?) and broader organisational context (i.e. what actions occurred and what decisions were taken in this environment) Provides a feel for organisational environments and the roles of actors within them that cannot be achieved through an interview situation.
Documentary analysis	PRE, DURING & POST	PRE: Meeting agendas and minutes Emails and telephone notes Draft operational plan Site decision making frameworks, policies, guides, tools, documentation developed by economist and sites including evidence of application DURING: Meeting notes, agendas, minutes, and associated documentation developed by the economist and sites including economic evaluations drafted, evidence of application and practice change from meeting agendas, minutes and site generated documentation will be collected POST: During the semi-structured interviews, participants will be asked to provide any documentation that demonstrate a change in processes and work practices as a result of the embedded Economist Project. Documentation may include meeting notes, agendas, minutes, policies, tools and guidelines	Site Leads and Economists and staff who planned or participated in the embedded Economist intervention will be asked to provide documents	Maximum 10 per site	2 h maximum (sourcing and providing documents)	Documentary evidence of how each stage of the intervention was formally enacted by the site and does this accord with what was observed and reported? Existing decision-making frameworks will also be analysed to ascertain the use of economic evaluation. Documentary analysis provides additional insights about the practices and around decision making and any practice outcomes from the intervention, thereby supporting any reported changes from interview data with tangible evidence
Semi-structured interviews (face-to-face or telephone)	PRE, DURING & POST	Interview transcripts and recordings	Economists and staff who planned or participated in the program	*PRE:* 5 staff 2 economists *DURING:* *25 staff* 2 economists *POST:* 5 staff 2 economists	maximum 1 h per interview (N.B staff participants may be interviewed once during each phase of the intervention & economists may be interviewed up to 6 times during each phase)	Face to face anonymous inquiry providing in-depth descriptions and interpretations that are constructed and separated from the time (and place) in which the events take place about the process, context and relational aspects of each stage of the embedded component of intervention, including planning, how the intervention was enacted and the outcomes and impact in the post phase; as well as the learning support components at the time of the semi-structured interview. Participants will have the opportunity to review their transcript prior to data analysis.
Field diary	PRE, DURING & POST	Field diary from economists & staff	Economists and staff who planned or participated in the program	PRE: Lead economist DURING: Lead and embedded Economist 25 staff	Staff: Minimum commitment 15 min per week × 12 weeks maximum commitment 1 h per week × 12 weeks) Economist: Minimum commitment 15 min per week × 15 weeks)	Reflections on how participants perceived and experienced each phase of the intervention. What did they do (process)? Who did they interact with (relational)? What broader organisational factors impacted each phase?
Education survey ** (DETAIL BELOW TABLE)	POST Completion of the university course	Online anonymous survey		Maximum of 60 across all sites		How are participants applying the knowledge gained from the university course in practice? Do they feel supported by their leadership to use evidence from economic evaluations? How are the learning supports contributing to practice change? i.e. have they increased capacity?

### Surveys

#### Baseline survey*

Current or emerging executives or managers who make decisions to improve the value of the programs, services, initiatives or technology for which they are responsible and who enrol in the ComPrac will be invited to participate in a 15 min anonymous online survey capturing a baseline measure of individual values and confidence and organisational support and barriers around economic evaluation; as well as expectations about the embedded Economist learning support component.

The survey is a modified version of two survey instruments previously implemented in Australia; one by Brennan and colleagues [24] and the other by Baghbanian and colleagues [25]. The Brennan survey measures the value an individual places on using research, confidence an individual has in their own knowledge and skills, knowledge an individual has of health evaluation, and value the organisation places on using research [24]. The Baghbanian survey was based on an internationally validated instrument (EUROMET) [11, 12] and measures the tools and systems an organisation has to support research engagement actions and use and barriers to the use of economic evaluations [25]. Additional questions to guide the ComPrac were formulated by the researchers. The results of the baseline survey will be used by the eE Program to tailor their embedded experiences at the sites yet to host an economist, and inform the management and content of the Community of Practice facilitated discussions.

#### Education component survey**

All site staff who complete the university course will be invited to undertake a 15 min anonymous online survey, based on a survey developed and implemented by Serrano and colleagues (2019) [26] . To increase response rates, participants will receive a maximum of four weekly email reminders, including a final email reminder.

In addition to background characteristics and demographic information, the survey questions cover the frequency and use of Course materials, knowledge and skills gained from the Course, reasons for not using Course materials and resources as much as intended, benefits from attending the course, leadership support for using evidence based evaluation data and tools, and perception of the influence of the Course in building capacity for economic evaluation.

Based on our capped university course recruitment numbers (60) we expect about 25 survey responses across all sites.

The researchers have also included specific questions in our interview schedule about the course and community of practice which will be asked during site evaluations.

### Data analysis

The surveys will be analysed for each individual site by calculating frequencies and descriptive statistics. Results of the site surveys will then be compared across sites for similarities and differences.

Interviews will be professionally transcribed. Participants will be invited to review their de-identified transcripts and request amendments before they are entered into QSR International’s NVivo qualitative data analysis software (Version 12).

Documents provided by the sites, field diaries and open survey questions as well as researcher observations will also be entered into NVivo. A six-stage hybrid thematic analysis technique consisting of a data-driven inductive approach and a template code deductive approach will be used to analyse interviews, observations, documents and field diaries, as described by Fereday and Muir-cochrame [27] and applied more recently by Yukhymenko et al. [28].

- Stage 1: involves the development of a code manual based on the interview guide, the research questions and the literature.
- Stage 2: involves testing and refining the manual and coding. Four of the researchers will each blind code three interviews and refine the manual and coding based on a comparison of their coding of these initial interviews.
- Stage 3: involves summarising the raw data in tabular form to derive more detailed sub-codes directly from the interviews.
- The next steps of data analysis will occur concurrently, including iterative and reflexive processes.
- Stage 4 and 5: involves applying the code manual to the remaining transcripts and connecting themes to identify similarities and differences between participants.
- Stage 6: the coded themes will be corroborated and legitimated by scrutinising the previous stages to ensure that clustered themes were representative of the initial data analysis and assigned codes.

Evaluation data collected at each site will be collated and analysed to identify similarities and differences across sites, including process, context and relational differences highlighting what works for whom and when, as well as broad themes, to inform recommendations for post project pathways to implementation at each site, and program spread.

Finally, to capture the impact of the overall program we will apply the Framework to Assess the Impact from Translational health research (FAIT) [29] to each project intervention. Locally developed, the FAIT assessment tool was selected as it is based on an extensive review of existing impact frameworks; and it emphasises translational health research and is therefore well suited to this research evaluation [29, 30].

FAIT combines the three most commonly used approaches to impact assessment:

- *Domains of benefit*: including knowledge generation, impacts on policy, clinical practice, health services or population health, and economic benefits.
- *Economic analysis*: that compares costs (of the research evaluation itself and of implementing recommendations), to social, clinical/environmental and economic consequences (expressed in monetary terms where possible) that flow from implementation; and
- *Case studies*: short narratives that provides a summary of how translation occurred and how research impact was generated. The text is structured around common sub-headings (need, research/evaluation response, outcome, impact lessons) and its purpose is to contextualise findings and explain outcomes.

## Discussion

In contrast to clinical interventions, the evaluation of organisational interventions in healthcare is often neglected [31, 32]. While it is likely to be valuable to utilise economic evaluation more extensively at the local level in healthcare, to date the benefit has not been conclusively established [33].

Our protocol for evaluating the eE Program describes a ‘slow science’ research design [21], designed to overcome this and other barriers to the use of economic evaluation listed in previous reports [17, 34–41]. As far as the authors are aware, no previous designs have sought to document and tease apart the process of interaction and the relational aspects of engagement of an embedded economist with health service managers.

The eE program design is based on findings from national research examining how to improve the local level of evaluation of healthcare in Australia [3]. While the national report provided four high level recommendations to build capacity and capacity in evaluation, selected aspects have been rapidly operationalised by the eE program. The eE intervention places health economists in a health services and delivers and provides an educational intervention tailored to the capacity building needs of decision makers at local level.

Around this intervention a sophisticated multi-site multi-method evaluation has been designed to assess the benefits gained from the investment in the eE Program. The major challenge of this study will be working with rich data from multiple sites over time, where the processes of co-production are highly influenced by context. Assuming a positive finding, defined as the benefit is viewed as being greater than the cost, the evaluation will also report information that will assist refinement of the eE program, translation and scale nationally. It will report what worked, what did not work, and what needs to be altered.

The eE program is a novel and ambitious research project to build useful capacity in a skill set that is in short supply for health services: health economics. Importantly its architecture was co-designed by end users in healthcare; it is those same end users the eE program is designed to benefit. If shown to be worth the investment, the eE program could be rolled out at scale. The structure and philosophy of eE program could also be extended to other health services skills (such as study design, epidemiology, biostatistics, data linkage, public health etc). It has the potential to be a national role model for embedding research expertise into local health services in a way that makes systematic work towards value-based care the norm rather than the exception.

## Supplementary Information

**Additional file 1.** Interview Guide for Staff participants.

**Additional file 2.** Interview Guide for embedded Economists.

**Additional file 3.** Baseline survey.

**Additional file 4.** Education survey.

## Data Availability

Not applicable.

## Abbreviations

AHRA: Australian Health Research Alliance
CCLHD: Central Coast Local Health District
CMN: Calvary Mater Newcastle
ComPrac: Community of Practice
eE: embedded Economist
FAIT: Framework to Assess the Impact from Translational health research
HMRI: Hunter Medical Research Institute
HNECCPHN: Hunter New England Central Coast Primary Health Network
HTSA: Health Translation South Australia
HNELHD: Hunter New England Local Health District
MNCLHD: Mid North Coast Local Health District
MRFF: Medical Research Future Fund
NHMRC: National Health and Medical Research Council
NSWRHP: New South Wales Regional health Partners
SALHN: Southern Adelaide Local Health Network

## References

[1] Australian Government (2015). Productivity commission: efficiency in health.

[2] Australian Institute of Health and Welfare (2019). Health expenditure Australia 2017–18.

[3] Searles A, Gleeson M, Reeves P, Jorm C, Leeder S, Karnon J, Hiscock H, Skouteris H, Daly M (2019). The local level evaluation of healthcare in Australia.

[4] Roseleur J, Partington A, Karnon J (2020). A scoping review of Australian evaluations of health care delivery models: are we making the most of the evidence?.

[5] Searles A (2009). The PBS in a globalised world: free trade and reference pricing. Aust Health Rev.

[6] Welfare AIoHa (2017). Health expenditure Australia 2015–16.

[7] Sorenson C, Japinga M, Crook H, McClellan M. Building A Better Health Care System Post-Covid-19: Steps for Reducing Low-Value and Wasteful Care. NEJM Catalyst Innov Care Deliv. 2020;1(4):1–10.

[8] Goodwin N. Understanding and evaluating the implementation of integrated care: A ‘three pipe’problem. Int J Integr Care. 2016;16(4):1–2. 10.5334/ijic.2609 PMC5354212 28316558

[9] Tsiachristas A, Stein KV, Evers S, Rutten-van Mölken M. Performing economic evaluation of integrated care: highway to hell or stairway to heaven? Int J Integr Care. 2016;16(4):1–12. 10.5334/ijic.2472 PMC5354211 28316543

[10] Ross J (1995). The use of economic evaluation in health care: Australian decision makers' perceptions. Health Policy.

[11] Hoffmann C, von der Schulenburg J-MG (2000). The influence of economic evaluation studies on decision making.: a European survey. Health Policy.

[12] Zwart-van Rijkom JE, Leufkens HG, Busschbach JJ, Broekmans AW, Rutten FF (2000). Differences in attitudes, knowledge and use of economic evaluations in decision-making in the Netherlands. Pharmacoeconomics.

[13] Buxton MJ (2006). Economic evaluation and decision making in the UK. Pharmacoeconomics.

[14] Baghbanian A, Torkfar G (2012). Economics and resourcing of complex healthcare systems. Aust Health Rev.

[15] Merlo G, Page K, Ratcliffe J, Halton K, Graves N (2015). Bridging the gap: exploring the barriers to using economic evidence in healthcare decision making and strategies for improving uptake. Appl Health Econ Health Policy.

[16] Merlo G, Page K, Zardo P, Graves N (2019). Applying an implementation framework to the use of evidence from economic evaluations in making healthcare decisions. Appl Health Econ Health Policy.

[17] Zechmeister-Koss I, Stanak M, Wolf S (2019). The status of health economic evaluation within decision making in Austria. Wien Med Wochenschr.

[18] Churruca K, Ludlow K, Taylor N, Long JC, Best S, Braithwaite J (2019). The time has come: embedded implementation research for health care improvement. J Eval Clin Pract.

[19] Vindrola-Padros C, Pape T, Utley M, Fulop NJ (2017). The role of embedded research in quality improvement: a narrative review. BMJ Qual Safety.

[20] Beckett K, Farr M, Kothari A, Wye L, le May A (2018). Embracing complexity and uncertainty to create impact: exploring the processes and transformative potential of co-produced research through development of a social impact model. Health Res Policy Syst.

[21] Stengers I. Another science is possible: a manifesto for slow science. New York: Wiley; 2018.

[22] Aveling EL, Martin G, Armstrong N, Banerjee J, Dixon-Woods M. Quality improvement through clinical communities: eight lessons for practice. J Health Organ Manag. 2012;26(2):158–74. 10.1108/14777261211230754 22856174

[23] Lave J, Wenger E. Situated learning: legitimate peripheral participation. Cambridge: Cambridge University Press; 1991.

[24] Brennan SE, McKenzie JE, Turner T, Redman S, Makkar S, Williamson A, Haynes A, Green SE (2017). Development and validation of SEER (seeking, engaging with and evaluating research): a measure of policymakers’ capacity to engage with and use research. Health Res Policy Syst.

[25] Baghbanian A, Hughes I, Khavarpour FA (2011). Resource allocation and economic evaluation in Australia’s healthcare system. Aust Health Rev.

[26] Serrano N, Diem G, Grabauskas V, Shatchkute A, Stachenko S, Deshpande A, Gillespie KN, Baker EA, Vartinaien E, Brownson RC (2020). Building the capacity–examining the impact of evidence-based public health trainings in Europe: a mixed methods approach. Glob Health Promot.

[27] Fereday J, Muir-Cochrane E (2006). Demonstrating rigor using thematic analysis: a hybrid approach of inductive and deductive coding and theme development. Int J Qual Methods.

[28] Yukhymenko MA, Brown SW, Lawless KA, Brodowinska K, Mullin G (2014). Thematic analysis of teacher instructional practices and student responses in middle school classrooms with problem-based learning environment. Global Educ Rev.

[29] Searles A, Doran C, Attia J, Knight D, Wiggers J, Deeming S, Mattes J, Webb B, Hannan S, Ling R (2016). An approach to measuring and encouraging research translation and research impact. Health Res Policy Syst.

[30] Dodd R, Ramanathan S, Angell B, Peiris D, Joshi R, Searles A, Webster J (2019). Strengthening and measuring research impact in global health: lessons from applying the FAIT framework. Health Res Policy Syst.

[31] Dixon-Woods M, Martin GP (2016). Does quality improvement improve quality?. Future Hosp J.

[32] Dixon-Woods M, McNicol S, Martin G (2012). Ten challenges in improving quality in healthcare: lessons from the Health Foundation’s programme evaluations and relevant literature. BMJ Qual Safe.

[33] Lessard C, Contandriopoulos A-P, Beaulieu M-D (2010). The role (or not) of economic evaluation at the micro level: can Bourdieu’s theory provide a way forward for clinical decision-making?. Soc Sci Med.

[34] Eddama O, Coast J (2008). A systematic review of the use of economic evaluation in local decision-making. Health Policy.

[35] Eddama O, Coast J (2009). Use of economic evaluation in local health care decision-making in England: a qualitative investigation. Health Policy.

[36] Lessard C (2007). Complexity and reflexivity: two important issues for economic evaluation in health care. Soc Sci Med.

[37] Hoffmann C, Stoykova BA, Nixon J, Glanville JM, Misso K, Drummond MF (2002). Do health-care decision makers find economic evaluations useful? The findings of focus group research in UK health authorities. Value Health.

[38] Roseboom KJ, van Dongen JM, Tompa E, van Tulder MW, Bosmans JE (2017). Economic evaluations of health technologies in Dutch healthcare decision-making: a qualitative study of the current and potential use, barriers, and facilitators. BMC Health Serv Res.

[39] Brousselle A, Lessard C (2011). Economic evaluation to inform health care decision-making: promise, pitfalls and a proposal for an alternative path. Soc Sci Med.

[40] Williams I, McIver S, Moore D, Bryan S. The use of economic evaluations in NHS decision-making: a review and empirical investigation. Health Technol Assess. 2008;12(7):1–196. 10.3310/hta12070 18373906

[41] Drummond M (2004). Economic evaluation in health care: is it really useful or are we just kidding ourselves?. Aust Econ Rev.

