# Supplementary material for: Embedding an economist in regional and rural health services to add value and reduce waste by improving local-level decision-making: protocol for the ‘embedded Economist’ program and evaluation
Source: BMC Health Serv Res. 2021 Mar 6;21:201. doi: 10.1186/s12913-021-06181-1 (PMC7936595; doi:10.1186/s12913-021-06181-1)
Supplement: Supplementary file 1 — Additional file 1. Interview Guide for Staff participants. [file 12913_2021_6181_MOESM1_ESM.docx]

**Interview Guide for Staff participants**

Planning

- *Skills:* How would you describe you/your site’s current economic evaluation skills?
- *Contextual:* How do you/ the site currently approach decision making? In particular how are economic evaluations incorporated? What are your/the site’s understandings about the role of the embedded Economist program?
- *Aims:* What are your aims from participating in this program individually/site?
- *Impact:* What do you perceive will be the main impact of the program at this site for you/your site?
- *eE:* What special skills and level of experience do you think are necessary for the embedded role to be successful at this site? Why?
- *Procedural:* How did you become involved in the program/ how did planning and recruitment occur at this site?
- *Pre-existing relationships:* Do you know the economist? Have you worked with them previously?
- *Nudging:* Does your site have set ideas on how the economist would embed during the pre-implementation phase? Or will you need to be guided by them?
- *Organisational factors*: Are there any organisational or political factors, i.e., aspects of readiness, culture, etc that you have experienced to date at play that will impact on the planning phase and/or program overall
- *Relational*: How do you feel about being involved in the embedded Economist Program? (Individually and overall site)
  - *Prompts*: What were the site’s motivations, perceptions and attitude towards the program? What value did the individual and the site place on developing staff’s knowledge, confidence and practice re economic evaluations? How did the individual/site perceive and experience the planning phase of the program? What benefits, if any, did they see for themselves and/or the organisation? What concerns, if any, did staff have for themselves and/or the organisation?
- *Barriers:* What have been the barriers to planning phase of the program? Please provide an example(s.) What would you do differently if the program was to be rolled out in another site?
- *Facilitators:* What have been the facilitators to the planning phase of the program? Please provide an example(s).
- *Other comments?*

Embedding

- *Aims:* What are your aims from participating in this project individually/site?
- *Impact:* What do you perceive will be the main impact of the program at this site for you/your site?
- *Contextual*: What contextual and/or site specific features effected embedding?
- *Organisational factors*: Are there any organisational or political factors, i.e., aspects of readiness, culture, etc that you have experienced to date at play?
- *Procedural*: How did you access the economist? What aspects of the embedding phase of the program did you participate in? Why/why not?
- *Relational*: How did participants perceive and experience the embedded phase?
  - *Prompts*: What benefits, if any, did they see and experience for themselves and/or the organisation in participating? What concerns, if any, did staff have for themselves and/or the organisation about the program and were these fears and anxieties realised? What happened when sites were confronted with a different way of doing things?
- *Chang*e: Has the program changed thinking and increased knowledge to date?
- C*ontrol, power and politics*: How does power and control play out; who sways decisions and how; why?
- *Barriers:* What have been the barriers to implementing the program? Please provide an example(s.)
- *Facilitators:* What have been the facilitators to implementing the program? Please provide an example(s).

Post –embedding

- *Aims and Impact:* Were your hopes for yourself and your organisation achieved? What were the main impacts of the program for you and your site?
  - *Prompt:* Has the eE changed thinking, ways of working and increased knowledge and capacity to date? Please provide examples. How did your site utilise the embedded Economist to generate the benefits they were looking for? Can you provide any examples that demonstrates where and how staff used the skills gained from the program?
- *Individual skills* *and attributes*: Have your economic evaluation skills increased as a result of the eE program? If so how? If not what could have been done differently to ensure they were?
- *Organisational capacity building*: Has your organisation’s economic evaluation skills increased as a result of the eE program? If so how? If not what could have been done differently to ensure they were?
- *Economist skills* *and attributes*: With the benefit of hindsight, what special skills and level of experience do you think are necessary for the embedded role to be successful?
- *Exiting*: How as the program finalised at this site?
- *Work done/Process:* What are the next steps for you and your site in relation to building/sustaining capacity n economic evaluation?
- *Sustainability:* Can the benefits of the program be sustained? Why/why not?
- *Spread:* What is the potential for spread of skills/ ways of working to other sites?
- *Organisational factors*: Were there any organisational or political factors, i.e., aspects of readiness, culture, etc that impacted on the program? If so how?
- *Barriers:* What have been the barriers to implementing the program? Please provide an example(s.) What would you do differently if the program was to be rolled out in another site?
- *Facilitators:* What have been the facilitators to implementing the program? Please provide an example(s).
- *Other comments?*

Education component

- Are you participating in the ComPrac and/or course on economic evaluation?

If yes:

- - How did you find it?
  - How are you applying the knowledge gained, in practice?
  - Do you feel supported by your leadership to use evidence from economic evaluations?
  - How is the ComPrac/course contributing to decision making and practice change?

If no:

- - Do you intend to enrol? Why/Why/not?
