# Supplementary material for: Embedding an economist in regional and rural health services to add value and reduce waste by improving local-level decision-making: protocol for the ‘embedded Economist’ program and evaluation
Source: BMC Health Serv Res. 2021 Mar 6;21:201. doi: 10.1186/s12913-021-06181-1 (PMC7936595; doi:10.1186/s12913-021-06181-1)
Supplement: Supplementary file 2 — Additional file 2. Interview Guide for embedded Economists. [file 12913_2021_6181_MOESM2_ESM.docx]

**Interview Guide for embedded Economists**

Planning phase

- *Aims:* What are your hopes for the project overall/at this site?
- *Impact:* What do you perceive will be the main impact of the program at this site?
- *Skills* *and attributes*: What special skills and level of experience do you think are necessary for the embedded role to be successful at this site? Why?
- *Contextual and procedural*: How did planning and recruitment occur at this site? How might the planning and recruitment phase be improved for this and future sites?
- *Assumptions and perceptions:* Based on your interaction to date, what are your assumptions about how this site makes decisions? How important do you think economic evaluation is to their decision making? Please comment on the site’s value, confidence, knowledge and use of health evaluation?
- *Organisational factors*: Are there any organisational or political factors, i.e., aspects of readiness, culture, etc that you have experienced to date at play that will impact on the program?
- *Relational:* How did you perceive and experience the planning phase of the program? What were you asked? Who did you engage with and who engaged with you?
- *Pre-existing relationship*: What is your relationship to the site? How do you think this has and will influence the program?
- *Barriers:* What have been the barriers to planning stage of the program? Please provide an example(s.) What would you do differently if the program was to be rolled out in another site?
- *Facilitators:* What have been the facilitators to the planning stage of the program? Please provide an example(s).
- *Other comments?*

Embedding phase

- *Aims:* What are your hopes for the project overall/at this site?
- *Impact:* What do you perceive will be the main impact of the program at this site?
- *Contextual*: What contextual and/or site specific features effected embedding?
- *Organisational factors*: Are there any organisational or political factors, i.e., aspects of readiness, culture, etc that you have experienced to date at play?
- *Procedural*: How did embedding occur at each site?
  - *Prompts*: How was the economist embedded within the site? i.e. What did the economists do during the embedded phase including: what projects did they work on, with whom? How much time did they spend on each project? On site? Off-site? Where they were physically located when on site? What formal activities did they arrange? Did this vary from what was decided in the planning phase? If so how?
- How did the site enact the balance between the embedding and education components of the program?
- How might the embedding phase be improved for this and future sites?
- *Relational*: How did the economist perceive and experience the embedding phase?
  - *Prompts*: What were the ways of working the economist enacted whilst embedded? What benefits, if any, did they see and experience for themselves and/or the organisation in participating? What concerns, if any, did economists have for themselves and/or the organisation about the program and were these fears and anxieties realised? What did the economist think were the characteristics required by an embedded economist and the organisation to ensure the success of the program? How did any pre-existing relationships with this site impact on embedding?
- *Chang*e: what happened when sites were confronted with a different way of doing things? Has the program changed thinking and increased knowledge/capacity re evaluation to date?
- C*ontrol, power and politics*: how does power and control play out: who sways decisions and how; why?
- *Barriers:* What have been the barriers to implementing the program? Please provide an example(s.) What would you do differently if the program was to be rolled out in another site?
- *Facilitators:* What have been the facilitators to implementing the program? Please provide an example(s).

Post – embedding

- *Aims and Impact:* Were your hopes for the program at this site achieved? What were the main impacts of the program? Has the eE changed thinking and increased knowledge to date? Please provide examples.
- *Skills* *and attributes*: With the benefit of hindsight, what special skills and level of experience do you think are necessary for the embedded role to be successful?
- *Capacity building*: How did the site utilise the embedded Economist to generate the benefits they were looking for? In your opinion, did staff believe the program had increased their understanding of economic evaluation? Their capacity to source and undertake it now /in the future? If so what aspects of it? If not why not? In your opinion has the program changed the way staff work? If so how? If not why not? Can you provide any examples that demonstrates where and how staff used the skills gained from the program? How has the site applied what they learned from the program?
- *Exiting*: How did you finalise the project and exit this site?
- *Work done/Process:* What are the next steps post the eE Program for this site?
- *Sustainability:* Can the capacity built be sustained? Why/why not?
- *Spread:* What is the potential for spread of skills/ways of working to other sites?
- *Barriers:* What have been the barriers to the program? Please provide an example(s.) What would you do differently if the program was to be rolled out in another site?
- *Facilitators:* What have been the facilitators for the program? Please provide an example(s).
- *Other comments?*
