# Supplementary material for: Embedding an economist in regional and rural health services to add value and reduce waste by improving local-level decision-making: protocol for the ‘embedded Economist’ program and evaluation
Source: BMC Health Serv Res. 2021 Mar 6;21:201. doi: 10.1186/s12913-021-06181-1 (PMC7936595; doi:10.1186/s12913-021-06181-1)
Supplement: Supplementary file 3 — Additional file 3. Baseline survey. [file 12913_2021_6181_MOESM3_ESM.docx]

| **DEMOGRAPHICS** | **Which organisation do you work for?**  Hunter New England Central Coast Primary Health Network (HNECCPHN)  Hunter New England Local Health District (HNELHD)  Mid North Coast Local Health District (MNCLHD)  Central Coast Local Health District (CCLHD)  Calvary Mater Newcastle (CMN)  Southern Adelaide Local Health Network (SALHN)  South Australia Health or related entity (SA Health or related)  Other (please specify below) | | | | | |
| --- | --- | --- | --- | --- | --- | --- |
|  | **How old are you?** | 18-25 | 26-35 | 36-45 | 46-55 | Over 55 |
|  | **What gender are you?** | Male | Female | Non-binary | Prefer not to disclose |  |
|  | **What is your decision making role within your organisation?** | Senior Executive | Executive | Health Service Manager Level 4-6 | Health Service Manager Level 1-3 | None of the above (Please specify) |
|  | **How long have you worked in the health service? *(Please select ONE answer)***  Less than 1 year  1 to 5 years  6 to 10 years  11 to 15 years  16 to 20 years  21 years or more | | | | | |
|  | **What is your professional background? *(Please select ONE answer that best describes your background)***  Nurse  Doctor  Allied Health Professional  Pharmacist  Accountant  Manager | | | | | |
| **VALUE (Individual)**  ***(Note the term "research"  is used broadly and includes "evaluation")*** | **In the context of my work, it is valuable to use research to:** | **not at all valuable** | **of low value** | **somewhat valuable** | **moderately valuable** | **very valuable** |
|  | Identify issues that should be a priority |  |  |  |  |  |
|  | Understand how to address priority issues |  |  |  |  |  |
|  | Persuade others to a point of view or course of action |  |  |  |  |  |
|  | Design the implementation or evaluation of a strategy or program of care |  |  |  |  |  |
|  | Monitor implementation or evaluate impact from a strategy or program of care |  |  |  |  |  |
|  | Meet organisational requirements to use research |  |  |  |  |  |
|  | Understand the costs and consequences of decisions about healthcare |  |  |  |  |  |
| **CONFIDENCE**  ***(Note the term "research"  is used broadly and includes "evaluation")*** | **Within the context of my health service, I feel confident that I have the knowledge and skills to:** | **not at all confident** | **not very confident** | **neither confident or unconfident** | **fairly confident** | **very confident** |
|  | Find relevant research |  |  |  |  |  |
|  | Evaluate the quality of research |  |  |  |  |  |
|  | Interpret the results of research |  |  |  |  |  |
|  | Apply research findings (e.g. in decision making) |  |  |  |  |  |
|  | Design evaluations |  |  |  |  |  |
|  | Commission research |  |  |  |  |  |
|  | Partner with researchers to generate research |  |  |  |  |  |
|  | Access economic advice when I need to |  |  |  |  |  |
|  | Commission an economic evaluation |  |  |  |  |  |
|  | Incorporate an economic evaluation into a decision-making process |  |  |  |  |  |
| **VALUE (Organisation)**  ***(Note the term "research"  is used broadly and includes "evaluation")*** | **In my organisation...** | **never** | **rarely** | **sometimes** | **frequently** | **always** |
|  | Leaders believe it is important to use research |  |  |  |  |  |
|  | It is expected that research will be used |  |  |  |  |  |
|  | Generation of new research relevant to my health service is encouraged |  |  |  |  |  |
|  | It is expected that programs, services, initiatives and technology will be evaluated |  |  |  |  |  |
|  | Interaction or collaboration with researchers or research organisations is encouraged |  |  |  |  |  |
| **TOOLS & SYSTEMS**  *(Note the term "research"  is used broadly and includes "evaluation")* | **My organisation…** | **no** | **yes but limited** | **yes well developed** | **I don’t know** |  |
|  | Has processes that provide guidance on how and when research should be used |  |  |  |  |  |
|  | Has systems that encourage leaders to support use of research |  |  |  |  |  |
|  | Provides access to training in using research |  |  |  |  |  |
|  | Has the resources needed to support research (e.g. ongoing subscriptions and access to journals, a library, relevant software, a research/evaluation team) |  |  |  |  |  |
|  | Has established methods for commissioning reviews of research |  |  |  |  |  |
|  | Has documented processes for how programs, services, initiatives and technology should be evaluated |  |  |  |  |  |
|  | Has existing relationships, or established methods for engaging with research organisations |  |  |  |  |  |
| **BARRIERS**  Here is a list of some factors identified in the literature that may discourage the use of the results of economic evaluations in healthcare decision making | **Please assess their relevance as barriers within your organisation:** | **not at all relevant** | **not very relevant** | **neither relevant or irrelevant** | **somewhat relevant** | **very relevant** |
|  | Savings/costs in economic studies are anticipated, not real |  |  |  |  |  |
|  | Economic studies make too many assumptions |  |  |  |  |  |
|  | Economic evaluations are not applicable to local context |  |  |  |  |  |
|  | Economic studies are clinically not relevant |  |  |  |  |  |
|  | Health economic reports are not easily understood by decision makers |  |  |  |  |  |
|  | Access to health economics skill sets is difficult |  |  |  |  |  |
|  | Lack of awareness of the economic evaluation approach to decision-making |  |  |  |  |  |
|  | Lack of time, money and other resources needed to conduct economic evaluations |  |  |  |  |  |
|  | Economic evaluations are used inappropriately to delay decisions |  |  |  |  |  |
|  | Other (please specify) |  |  |  |  |  |
| **OTHER COMMENTS** | **Please feel free to provide any other comments about *research and evaluation* you would like the researchers to consider here:** | | | | | |
| **EXPECTATIONS**  **We are interested in how you think** the embedded Economist online learning support **will impact the way you do your work. Please tell us about your expectations in relation to the following** | **I expect the embedded Economist online learning support will:** | **not at all** | **very little** | **undecided** | **somewhat** | **greatly** |
|  | Provide a welcoming, inclusive and responsive learning environment |  |  |  |  |  |
|  | Expand my collaborative network |  |  |  |  |  |
|  | Breakdown communication barriers between experts and health services staff |  |  |  |  |  |
|  | Enable me to take action on ideas that were generated as a result of my involvement |  |  |  |  |  |
|  | Showcase good practices I can apply to my work |  |  |  |  |  |
|  | Motivate me to share work-related issues |  |  |  |  |  |
|  | Increase my knowledge of research/health economics |  |  |  |  |  |
|  | Increase my capacity to apply evidence-based practices |  |  |  |  |  |
|  | Increase my capacity to meet organisational requirements |  |  |  |  |  |
| **ACCESS**  ***(Please select all that apply)*** | **Who would you like to be able to access in the embedded Economist online learning support**  Academic health economists  Applied health economists  Representatives from Government Treasury Departments  Representatives from Health Departments  Colleagues in a similar position to me from other health services  Other (please specify) | | | | | |
| **OTHER COMMENTS** | **Please feel free to provide any other comments about the embedded Economist online learning support you would like considered:** | | | | | |
