# Supplementary material for: Embedding an economist in regional and rural health services to add value and reduce waste by improving local-level decision-making: protocol for the ‘embedded Economist’ program and evaluation
Source: BMC Health Serv Res. 2021 Mar 6;21:201. doi: 10.1186/s12913-021-06181-1 (PMC7936595; doi:10.1186/s12913-021-06181-1)
Supplement: Supplementary file 4 — Additional file 4. Education survey. [file 12913_2021_6181_MOESM4_ESM.docx]

# **Education survey**

*Participants who complete the University of Newcastle course titled “Health Economics and Finance*” *will be asked the rate the following statements on a Likert scale, via an online survey link developed in Qualtrics and emailed to them via an anonymous link*

**DEMOGRPAHIC INFORMATION**

Age:

Gender:

Employer:

Job Description:

**FREQUENCY AND USE OF COURSE MATERILAS AND BENEFITS FROM ENROLLING**

*Survey participants will be asked to rate the following on a five-point Likert scale ranging from (seldom/never,*

*quarterly, monthly, weekly, daily*

**On average, every month since the course I have**

Referred to the readings that were provided

Used the materials/skills in planning a new programme/intervention

Used the materials/skills in modifying an existing programme/intervention

Used the materials/skills for grant applications

Used the materials/skills in searching the scientific literature for information on programmes/interventions

Used the materials/skills in evaluating a programme/intervention

Used the materials/skills to write up the results of a programme/intervention

Other (please specify)

**The course content helped me to**

Acquire knowledge about a new subject

See applications for this knowledge in my work

Make scientifically informed decisions at work

Communicate better with co-workers who use economic evaluation skills

Read scientific reports and articles

Obtain funding for programmes at work

Develop a rationale for a policy change

Identify and compare the costs and benefits of a programme or policy

Prepare reports for policymakers

Adapt an intervention to a stakeholder’s needs while keeping it evidence based

Teach others how to use/apply the information in the course

Become a better leader who promotes evidence-based decision-making

Other (please specify)

**REASONS FOR NOT USING COURSE MATERIAL**

*Survey participants will be asked to rate the following on a five-point Likert scale ranging from ‘strongly disagree to agree’*

I have not used the course content as much as I would like because

I do not have enough time to implement economic evaluation approaches

The information was too complex

The information lacked relevance

There was too much information and not enough time to process it

My organisation does not have a culture that supports the use of economic evaluation approaches

Within my unit, there are no incentives to use economic evaluation tools

There is not enough funding for continued training in economic evaluation

The people I work with do not have economic evaluation training

Other (please specify)

**LEADERSHIP SUPPORT**

*Survey participants will be asked to rate the following on a five-point Likert scale ranging from ‘Not at all supportive’ to ‘Extremely Supportive’*

The approach of leaders in my organisation to evidence from economic evaluation is

**CAPACITY BUILDING**

*Survey participants will be asked to rate the following on a five-point Likert scale ranging from ‘strongly disagree to agree’*

**Overall the course has**:

Changed my attitude about the use of economic evaluations

Increased my knowledge about economic evaluation methods and tools

Increased my confidence to apply economic evaluation methods and tools

Increased my usage of economic evaluation methods and tools

Other (please specify)

**OTHER COMMENTS**

Please feel free to provide any other comments about the course you would like the researchers to consider here:
